# Tumoricidal properties of thymoquinone on human colorectal adenocarcinoma cells via the modulation of autophagy

**DOI:** 10.1186/s12906-024-04432-2

**Published:** 2024-03-26

**Authors:** Mohammad Saleh Moulana, Sanya Haiaty, Ahad Bazmani, Roya Shabkhizan, Marziyeh Sadat Moslehian, Fatemeh Sadeghsoltani, Mostafa Mostafazadeh, Mohammad Reza Asadi, Mehdi Talebi, Zahra Jafari, Mohammad Reza Morovati, Mohammad Hosein Farzaei, Reza Rahbarghazi

**Affiliations:** 1 Department of Persian Medicine, Faculty of Medicine, Kermanshah University of Medical Sciences, Kermanshah, Iran; 2 Drug Applied Research Center, Tabriz University of Medical Sciences, Tabriz, Iran; 3 Department of Pathobiology, Faculty of Veterinary Medicine, Ferdowsi University of Mashhad, Mashhad, Iran; 4 Infectious and Tropical Diseases Research Center, Tabriz University of Medical Sciences, Tabriz, Iran; 5 Department of Medical Genetics, Faculty of Medicine, Tabriz University of Medical Sciences, Tabriz, Iran; 6 Department of Biochemistry and Clinical Laboratories, Tabriz University of Medical Sciences, Tabriz, Iran; 7 Stem Cell Research Center, Tabriz University of Medical Sciences, Tabriz, Iran; 8 Department of Applied Cell Sciences, Faculty of Advanced Medical Sciences, Tabriz University of Medical Sciences, Tabriz, Iran; 9 Pharmaceutical Sciences Research Center, Kermanshah University of Medical Sciences, Kermanshah, Iran; 10 Department of Traditional Pharmacy, Faculty of Pharmacy, Kermanshah University of Medical Sciences, Kermanshah, Iran

**Keywords:** Human colorectal cancer, Thymoquinone, Tumoricidal effects, Autophagy, Wnt/β-catenin pathway

## Abstract

Colorectal cancer (CRC) is deadly anaplastic changes in the gastrointestinal tract with high-rate mortality. In recent years, the application of phytocompounds has been extended along with different therapeutic protocols. Here, we monitored the effects of Thymoquinone (TQ) on autophagy via mitochondrial function after modulation of the Wnt/β-catenin signaling pathway.

Human colorectal adenocarcinoma HT-29 cells were treated with TQ (60 µM) and 15 µM Wnt3a inhibitor (LGK974) for 48 h. The survival rate was evaluated using an MTT assay. The expression of Wnt-related factors (c-Myc, and Axin), angiogenesis (VE-Cadherin), and mitophagy-related factors (PINK1, OPTN) was assessed using real-time PCR assay. Protein levels of autophagy factors (Beclin-1, LC3, and P62) were monitored using western blotting. Using flow cytometry analysis, the intracellular accumulation of Rhodamine 123 was evaluated. The migration properties were analyzed using a scratch wound healing assay.

Data indicated that TQ can reduce the viability of HT-29 cells compared to the control cells (*p* < 0.05). The expression of VE-Cadherin was inhibited while the expression of PINK1 was induced in treated cells (*p* < 0.05). Both LGK974 and TQ-treated cells exhibited activation of autophagy flux (Beclin-1↑, LC3II/I↑, and p62↓) compared to the control group (*p* < 0.05). TQ can increase intracellular accumulation of Rhodamine 123, indicating the inhibition of efflux mechanisms in cancer cells. Along with these changes, the migration of cells was also reduced (*p* < 0.05).

TQ is a potential phytocompound to alter the dynamic growth of human colorectal HT-29 cells via the modulation of autophagy, and mitophagy-related mechanisms.

**Supplementary Information:**

The online version contains supplementary material available at 10.1186/s12906-024-04432-2.

## Introduction

Colorectal cancer (CRC) is the third most common type of anaplastic changes in the gastrointestinal tract with debilitating consequences [1]. Statistical data have indicated that the morbidity rate of CRC has dramatically increased in recent years worldwide [2]. The rate of CRC is high in developed countries while its incidence is increasing in middle and low-income countries because of Westernization [2]. It is thought that several predisposing factors are involved in the pathology of CRC, indicating the multifactorial etiology of this cancer type.

Among these mechanisms, emerging findings have revealed the crucial role of autophagy in several cancer types such as malignant colorectal cells [3]. Under normal conditions, the activation of autophagy, as an evolutionarily conserved catabolic process, happens in response to several insulting conditions to restore the function of cells via the digestion of injured organelles and the elimination of misfolded proteins [4]. Despite its regenerative effects, it is suggested that autophagy is a double-edged sword in many types of human cancers, such as CRC because regulates the expression of various tumor suppressor and oncogenes genes [5]. Autophagy limits tumorigenesis and effectively the probability of CRC occurrence by removing damaged cellular organelles, reactive oxygen species, misfolded proteins, etc. [6]. Along with these descriptions, it is thought that autophagy can be considered as an alternative oncostatic strategy for preventing tumor formation and metastasis. On the other hand, autophagy can support the energy required for tumor cell proliferation through catabolic reactions under stressful conditions such as nutrient deficiencies, hypoxia, and cancer treatment protocols [6]. There is also evidence to suggest that mitophagy, selective autophagy of mitochondria, plays a role in CRC tumorigenesis [7].

It has been indicated that the Wnt/β-catenin pathway has an important role in maintaining the dynamic growth of tumor cells, drug resistance, and metastasis [8]. The connection between this signaling pathway and the autophagy process has been proven in various cancers [9]. In solid and non-solid tumors, the dysregulation of the Wnt/β-catenin signaling pathway can pre-determine cancer cell fate and tumorigenesis properties [10]. Abnormal Wnt/β-catenin signaling pathway activity in CRC is caused by a mutation in the tumor suppressor gene, leading to the activation of Wnt/β-catenin transduction signaling pathways involved in cell proliferation and progression [11].

In recent years, the application of natural compounds has increased in patients suffering from cancers [12]. In this regard, several in vivo and in vitro studies have used different natural compounds such as Curcumin, Bufalin, Ursolic acid, Thymoquinone (TQ), etc. for the treatment of CRC by targeting the autophagy pathway through different signaling pathways such as AMPK/ULK1, Wnt/β-catenin, and PI3K/AKT [13]. TQ, extracted from black seed oil (*Nigella sativa*), exhibits several biological effects such as hepato-protective, anti-inflammatory, and anticancer properties [14]. This compound can reduce CRC tumor cell growth and invasion by activating autophagic and apoptotic changes [15]. By inhibiting the NF-kB signaling pathway and pro-angiogenesis factors such as VEGF, TQ sensitizes the resistant CRC cells to chemotherapeutics like 5-Fluorouracil [16].

Despite the oncostatic effects of TQ, there are still conflicting debates on the role of TQ in regulating autophagy in cancer cells. Further investigations are needed to fully understand the molecular mechanisms by which TQ exerts its anticancer effects in CRC cells. Here, we aimed to clarify the possible relation between the effects of TQ on autophagy via mitochondrial function after modulation of the Wnt/β-catenin signaling pathway.

## Methods

### Cell culture protocol and expansion

The human colon cancer HT-29 cell line was obtained from the Pasteur Institute (Tehran, Iran). HT-29 cells were expanded using RPMI-1640 (Gibco) culture medium at standard conditions (37 °C, 5% CO_2_, 95% relative humidity). The culture medium was supplemented with 10% fetal bovine serum (FBS, Gibco) and 1% Penicillin/Streptomycin (Gibco), and changed every 3–4 days. Using 0.25% Trypsin-EDTA solution, cells were subcultured. Cells in passages 3–6 were used in this study for different experiments.

### MTT assay

To assess the viability of cells, the conventional MTT (Sigma-Aldrich) colorimetric method was used. HT-29 cells (∼ 1 × 10^4^) were placed in each well of 96-well plates (SPL, Korea) and maintained at standard conditions to reach 70–80% confluence. Then, cells were exposed to 60 µM TQ (Cat no: 15,039; Cayman; USA), 15 µM Wnt3a inhibitor (LGK974; Cat no: 14,072; Cayman; USA), and their combination for 48 h according to previously published data [16–18]. After treatment, supernatants were removed and 100 µl of 5 mg/ml MTT solution was overlaid on each well. Plates were maintained at 37 °C for 4 hours. In the next step, supernatants were replaced with 200 µl dimethyl sulfoxide (DMSO; Merck). The OD of each well was measured at 630 nm using a microplate reader. In this study, TQ and LGK974 stocking solutions were prepared using DMSO solution and a final concentration of solvent was below 1%.

### Real-time PCR analysis

The expression of genes related to mitophagy [PINK 1 and optineurin (OPTN)], WNT signaling pathway (Axin and c-myc), and VE-cadherin (an angiogenesis factor) was measured by real-time PCR technique. The total RNA of cells was extracted using Trizol Reagent (Cat no: 302-001; RiboExLs). RNA concentration was determined using NanoDrop® ND-1000 (NanoDrop Technologies, USA). The isolated RNAs (1 µg) were reverse transcribed into cDNA using a Takara cDNA Synthesis Kit (Cat no: RR037A). In this study, exon-exon junction spanning primers were designed for the specific detection of target genes (Table 1). The amplification process was performed in a MIC real-time PCR system (BioMolecular Systems, UK) using a program for 40 cycles as follows; denaturation for 10 s at 95 °C, annealing for 30 s at optimized annealing temperature (see Table 1) for each primer, and extension for 20 s at 72 °C. To run the reaction, a solution consisting of 1 µl of diluted (0.5 µM) primers (either forward or reverse sequence), 7 µl SYBR green DNA PCR Master Mix 2X (Ampliqon), 4 µl nuclease-free H_2_O_2_, and 1 µl cDNA samples. Fold changes of genes were calculated by the 2^−ΔΔCT^ method normalized to β-actin as a reference gene.

**Table 1:** Primers list

Gene	Forward	Reverse	TM (ºC)
Axin-2	5´-GACGGACAGCAGTGTAGATG-3´	5´-CGGGAAATGAGGTAGAGACAC-3´	58
c-myc	5´-CAGCGACTCTGAGGAGGAAC-3´	5´-GCGTAGTTGTGCTGATGTGTG-3´	59.5
VE-cadherin	5´- CCAGCAAAAGCAGGGAGTCTGT-3’	5’- TGTCTGTGTCATCGGAGTGATATCC-3’	58
OPTN	5´-CAGCGGCTCCTCAGAAGATT-3´	5´-GGCCCAGGACTATGCTTGAT-3´	60
PINK1	5´-TTTGCCCCTAACACGAGGAA-3´	5´-AACTGAACGTGCTGACCCAT-3´	60
β-actin	5´-TCCCTGGAGAAGAGCTACG-3´	5´-GTAGTTTCGTGGATGCCACA-3´	58

### Western blotting analysis

Western blotting was performed to measure protein levels of autophagy. After a 48-hour incubation period, cells were collected and lysed using a lysis buffer solution [1% NP-40, 50 mM Tris-HCl, EDTA, 150 mM NaCl, 0.5% sodium deoxycholate, and 1 mM protease inhibitor cocktail]. Cells were centrifuged at 12,000 g for 30 min at 4 °C. Samples were electrophoresed at 10% SDS-PAGE gel and transferred onto the PVDF membrane. Membranes were blocked with 2% skim milk at RT for one hour and exposed to primary antibodies solution at 4 °C overnight: Beclin-1 (Cat no: sc-48,341; Santa Cruz Biotechnology, Inc.), P62 (Cat no: sc-10,117; Santa Cruz Biotechnology, Inc.), and LC3 (Cat no: 2775; Cell Signaling Technology, Inc.). After several PBST washes, membranes were incubated with the secondary HRP-conjugated anti-IgG antibodies (Cat no: sc-2357; Santa Cruz Biotechnology, Inc.). Target immunoreactive bands appeared on the X-ray films using an ECL solution. Band density was calculated using ImageJ software (NIH) related to an internal housekeeping control protein β-actin (Cat no: sc-47,778 Santa Cruz Biotechnology, Inc.).

### Flow cytometric analysis of Rhodamine123 efflux capacity

HT-29 cells (3 × 10^5^ cells/well) were seeded in six-well plates. On 70–80% confluence, cells from different experimental groups were detached using Trypsin-EDTA and incubated with 1 µg/ml Rhodamine 123 (Cas no: 62,669–70 − 9; Sigma-Aldrich) 37 °C for 40 min. After that, cells were washed twice with PBS, resuspended in 500 µl ice-cold PBS, and analyzed with a flow cytometer system (BD Biosciences, USA) and FlowJo software (Ver. 7.6.1).

### Migration assay

Cells were seeded in six-well plates to generate a single-cell monolayer. To induce a scratch line, a sterile 100-µl pipette tip was used. After washing with sterile PBS, cells were treated with 60 µM TQ with or without LGK974 and incubated in a culture medium for another 48 h. Images were taken using an inverted microscope (Optika, Italy) at 0, 24, and 48 h post-wounding, and the distance between the scratch edges was measured using ImageJ software.

### Statistical analysis

Data (mean ± SD) were monitored using One-Way ANOVA with Tukey post hoc analysis. *p* < 0.05 was considered statistically significant.

## Results

### TQ and LGK974 reduced the viability of HT-29 cells

**Fig. 1:**
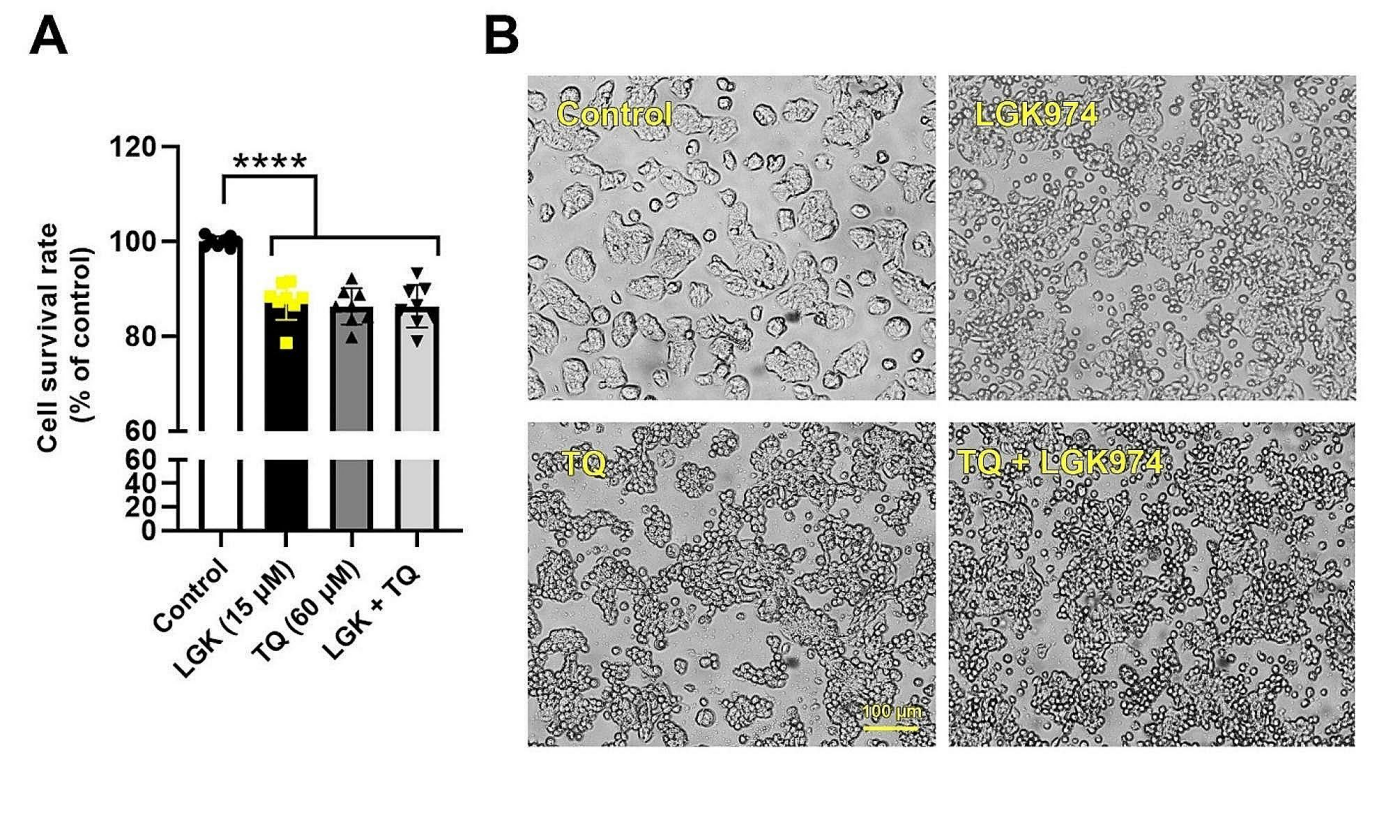
MTT assay. The viability of human colorectal adenocarcinoma HT-29 cells was monitored using an MTT assay after 48 h (**A**; *n* = 7). Data indicated a significant reduction of HT-29 cell viability after being exposed to LGK974 (Wnt3a inhibitor) and TQ. Compared to the TQ and LGK974 groups, treatment of cells with the combination of TQ and LGK974 did not yield statistically significant results. Morphological features of cells after being treated with TQ, LGK974, and TQ + LGK974 (**B**). In the treated groups, loosened cell-to-cell connections led to the appearance of numerous round-shaped single cells. One-way ANOVA and Tukey post hoc test. ****p < 0.0001

The survival rate was measured in HT-29 cells treated with 60 µM TQ, and 15 µM LGK974, and their combination after 48 h (Fig. 1A). Data exhibited an inhibitory activity of 60 µM TQ on HT-29 cells compared to the control group (*p*_*Control* Vs. TQ_<0.001). Similarly, 48-hour treatment of HT-29 cells with LGK974 led to a significant reduction of survival rate as compared to the control cells (*p*_*Control* Vs. LGK974_<0.001) (Fig. 1A). Likewise, the combination of TQ, and LGK974 decreased the viability of HT-29 cells related to non-treated control cells. Of note, non-significant differences were found in terms of survival rate in cells exposed to the combination of TQ and LGK974 compared to TQ and LGK974 groups (*p* > 0.05). Bright-field images revealed the existence of numerous spherical aggregates composed of a multilayer of cells (Fig. 1B). Bright-field imaging showed morphological changes in HT-29 cells after treatment with TQ, LGK974, and their combination after 48 h. Numerous single cells with round-shape morphologies were detected at the surface of plates in all these groups. These data indicated that blocking Wnt3a and treatment with TQ can loosen the connection between human adenocarcinoma HT-29 cells in in vitro conditions.

### Real-time PCR analysis

**Fig. 2:**
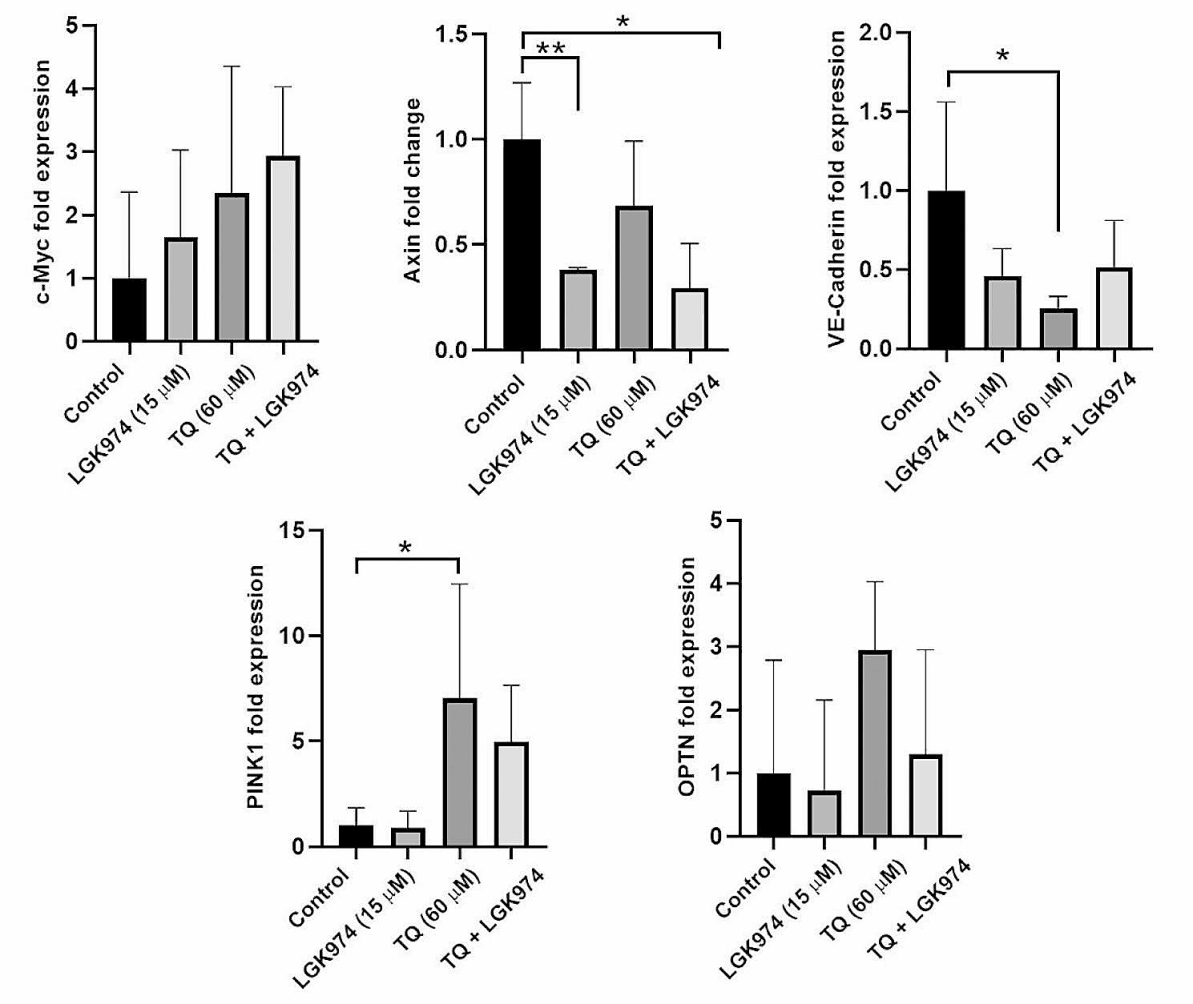
Real-time PCR analysis of different genes in HT-29 cells 48 h after treatment with LGK974 and TQ, and their combination. The expression of genes related to the Wnt signaling pathway (c-Myc and Axin), angiogenesis (VE-Cadherin), and mitophagy-related factors (PINK1, OPTN) were studied in HT-29 cells. One-way ANOVA and Tukey post hoc test (*n* = 3). *p < 0.05

Real-time PCR analysis indicated treatment of HT-29 cells with 60 µM TQ, 15 µM LGK974, and TQ + LGK974 did not affect the expression of c-Myc after 48 h (*p* > 0.05; Fig. 2). Despite the increase in c-Myc transcription, non-significant differences were obtained. Of note, we found that the treatment of HT-29 cells with LGK974, and TQ + LGK974 led to the down-regulation of Axin compared to the control cells (*p* < 0.05; Fig. 2). Data indicated that TQ did not reduce the expression of Axin. The expression of VE-cadherin was also monitored to indicate the angiogenesis potential. Data revealed the down-regulation of VE-cadherin in LGK974, TQ, and TQ + LGK974 groups however these values were statistically significant in TQ related to the non-treated cells. To monitor mitophagy response, we measured the transcription of levels of PINK1 and OPTN in HT-29 cells after 48 h. Of note, data showed that the expression of the PINK1 gene is increased in TQ, TQ-treated cells as compared to the control cells (*p* < 0.05; Fig. 2). Despite the increase of PINK1 in the TQ + LGK974 group, these values did not reach a statistically significant difference. The expression of OPTN was also increased in TQ, and TQ + LGK974 groups but did not reach statistically significant differences. These features demonstrate that TQ can induce the expression of mitophagy-related genes and diminished angiogenesis potential via the reduction of VE-cadherin.

### TQ and LGK974 induced autophagy response in HT-29 cells

**Fig. 3:**
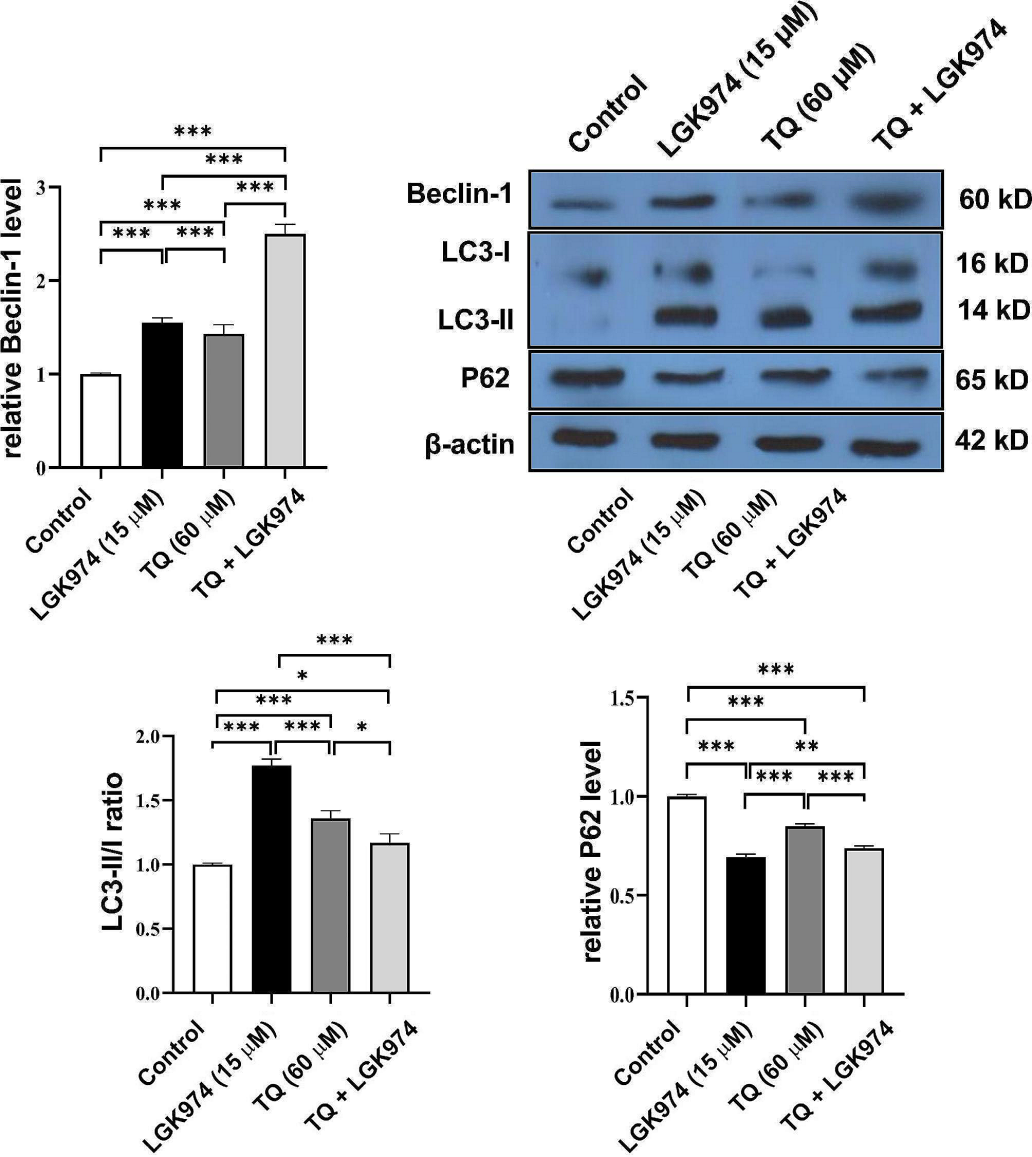
Monitoring autophagy-related proteins (Beclin-1, LC3-II/I ratio, and P62) in HT-29 cells after treatment with Wnt3a inhibitor (LGK974), TQ, and LGK974 + TQ. Data indicate the induction of autophagy response in HT-29 cells indicated by the increase of Beclin-1 and LC3-II/I ratio and reduction of P62 in the treated groups. One-way ANOVA and Tukey post hoc test (*n* = 3). *p < 0.05, **p < 0.01; ***p < 0.001

To investigate the effect of TQ on autophagy response in HT-29 cells, protein levels of LC3, P62, and Beclin-1 were monitored using western blotting after 72 h (Fig. 3). The data indicated that treatment of HT-29 cells with 60 µM TQ, 15 µM LGK 974, and TQ + LGK974 led to induction of autophagy via the increase of Beclin-1 compared to the non-treated control group (*p* < 0.05; Fig. 3). These effects were high in the LGK974 + TQ compared to the LGK974 and TQ groups. Data revealed the lack of statistically significant differences in terms of Beclin-1 levels between the LGK974 and TQ groups. We found that TQ, LGK974, and TQ + LGK974 promoted autophagy flux in HT-29 cells after 48 h indicated by an increased LC3-II/I ratio compared to the non-treated control cells (Fig. 3). Based on the western blotting panel, LGK974 exhibited the highest effect on the conversion of LC3-I into LC3-II. Compared to the LGK974 group, TQ alone increased significantly the LC3-II/I ratio. Data revealed the reduction of P62 levels in Ht-29 cells after being treated with TQ, LGK974, and TQ + LGK974 compared to the control cells (Fig. 3). We found maximum effects in the LGK974 group compared to TQ and TQ + LGK974 groups. These data indicate that treatment of human HT-29 cells with TQ, LGK974, and TQ + LGK974 can promote the autophagy signaling by the increase of Beclin-1, and LC3-II/I ratio while P62 reduction indicates the completion of autophagy response in these groups.

### Treatment with TQ and LGK974 reduced the efflux capacity of tumor cells

**Fig. 4:**
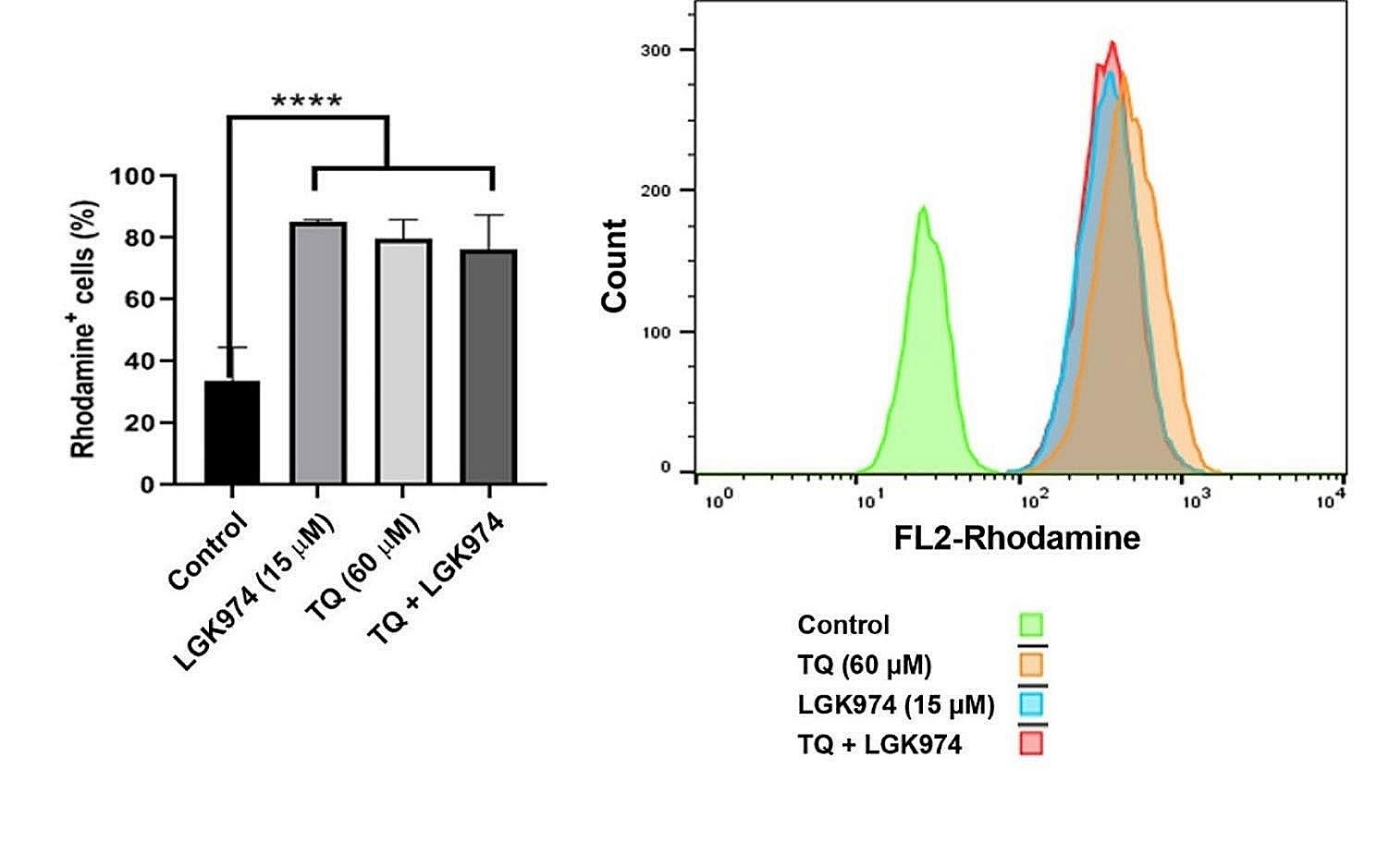
Monitoring HT-29 cell efflux capacity using Rhodamine 123 after being exposed to TQ, LGK974, and their combination. TQ, LGK974, and TQ + LGK974 can increase the intracellular accumulation of Rhodamine 123 inside the cells after 48 h compared to the non-treated control group. One-way ANOVA and Tukey post hoc test (*n* = 3). ****p < 0.0001

Flow cytometry data indicated that 48-hour incubation of HT-29 cells with TQ, LGK974, or their combination led to intracellular accumulation of Rhodamine 123 (Fig. 4). Compared to non-treated control cells, the fluorescence intensity of TQ, LGK974, and TQ + LGK974 treated cells increased significantly (*p* < 0.0001; Fig. 4). We found statistically non-significant differences in terms of fluorescence intensity between treated groups (*p* > 0.05). These features indicate that the exposure of HT-29 cells with TQ, LGK974, and their combination can abrogate the efflux capacity.

### TQ, but not LGK974, inhibited HT-29 cell migration

**Fig. 5:**
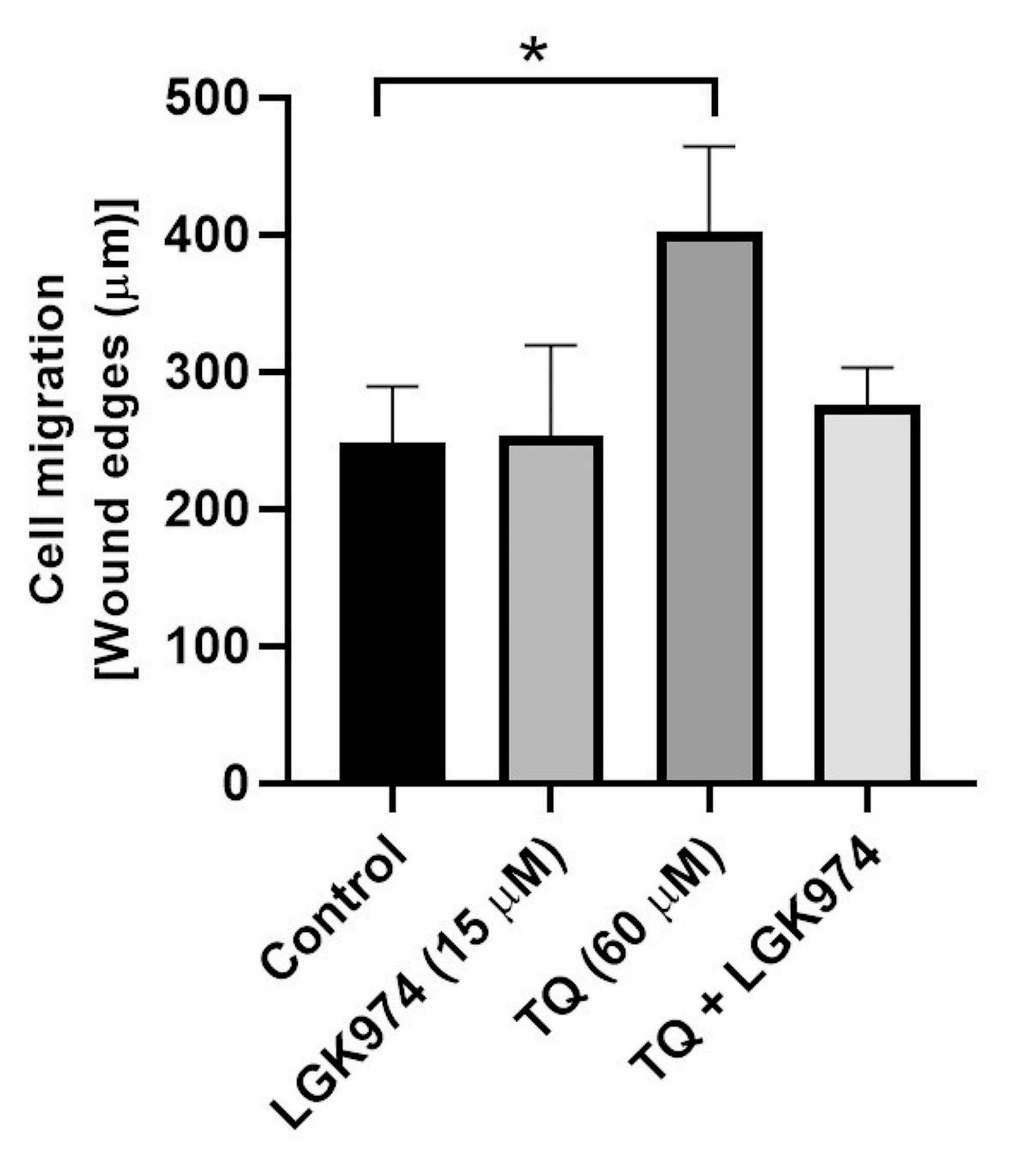
Scratch-wound assay was used to assess the migration of HT-29 cells in in vitro conditions after 48 h. Data showed an efficient inhibitory effect of TQ in the control of HT-29 cell migration compared to the control cell. One-way ANOVA and Tukey post hoc test (*n* = 3). *p < 0.05

The scratch-wound assay was performed to assess the inhibitory effect of TQ, LGK974, and their combination on human HT-29 cells after 48 h in in vitro conditions (Fig. 5). Data indicated that 15 µM LGK974 did not affect the migration of HT-29 cells in which the distance between the scratch edges remained statistically unchanged compared to the control group (248.1 ± 41.3 versus 253.2 ± 66 μm; *p* > 0.05) (Fig. 5). While in TQ treated cells, the distance between scratch edges was at the maximum levels compared to other groups especially non-treated control cells (248.1 ± 41.3 versus 402.1 ± 62.1 μm *p* < 0.05). Despite a slight increase of scratch edges in the TQ + LGK974 group (275.7 ± 27.4 μm), no statistically significant differences were found as compared to the control and other experimental groups (*p* > 0.05). These data indicated that TQ alone can efficiently affect the migration of HT-29 cells in in vitro conditions.

## Discussion

In terms of clinical applications, chemotherapeutics exhibit remarkably tumoricidal effects against different tumor cell types [19]. Nevertheless, chemotherapy-based approaches can lead to several side effects and even the emergence of resistant tumor cells in patients undergoing treatment protocols [20, 21]. Along with various chemical agents, a wide range of phytocompounds are currently being used as potent natural substrates for the prevention/treatment of cancers such as CRC [22–24]. Despite the conduction of several studies related to the anti-tumor properties of TQ, the exact underlying mechanisms remain unknown. Here, in this study, the possible modulatory effect of TQ on autophagy response and its relationship with the dynamic growth of human adenocarcinoma HT-29 cells were examined in vitro.

Present data indicated that 48-hour incubation of human HT-29 cells with TQ reduced the survival rate compared to control cells. Several experiments have indicated the anti-tumor properties of TQ against multiple cancer cells and in different animal models [25]. The inhibition of cell proliferation, metastasis, angiogenesis, invasion, promotion of apoptosis, and cytotoxicity have been proved in tumor cells originating from the liver, pancreas, breast, colorectal, blood, cervix, etc. [26–28]. The tumoricidal properties of TQ were recently shown by this research group on breast cancer stem cells by the inhibition of vascularization [29]. Consistent with our previous study, here we indicated that TQ alone can reduce the orientation of colorectal HT-29 cells toward endothelial lineage. To be specific, one possible anti-tumor properties of TQ are associated with the regulation of tumor cell differentiation toward endothelial cells and angiogenesis. It seems that the combination of TQ with conventional chemotherapeutics can increase anti-tumor potential. For instance, TQ in combination with docetaxel can efficiently block PI3K/AKT signal pathways and reduce the viability of DU-145 prostate cancer cells [30].

Besides, the roles of survival and proliferation mechanisms in cancer development and propagation, autophagy, as early-stage cell bioactivity, can blunt the efficiency of chemotherapeutics [31]. Therefore, the modulation of autophagy is an appropriate strategy to sensitize cancer cells against therapeutic protocols [32–34]. Here, we found that the exposure of HT-29 cells to Wnt3a inhibitor TQ induced autophagic response (Beclin-1↑, LC3II/I↑) alone or in combination compared to the control group. In line with the induction of autophagic flux, the levels of intracellular p62 were reduced in TQ, LGK974, and TQ + LGK974 treated cells, indicating the completion of autophagy response. Despite the stimulation of autophagy machinery in treated cells, the survival rate was decreased, indicating the harmful effects of autophagic response in these cells. In support of this notion, the expression of mitophagy-related factor PINK1 was significantly up-regulated in TQ treated group, indicating the activation of autophagy response via mitochondrial injury (mitophagy). Concurrently, flow cytometry analysis revealed the intracellular accumulation of Rhodamine 123 and efflux mechanism insufficiency in treated cells. Therefore, one can hypothesize that the induction of mitochondrial injury and subsequent autophagic response can lead to tumoricidal outcomes following treatment with TQ. Consistent with current data, previous data showed that TQ can stimulate autophagy and has a significant cell death-inducing effect mechanism in docetaxel-resistant prostate cancer cells [35]. Under stressful conditions, the activation of certain kinases such as DAPK and JNK1/2 contributes to the phosphorylation of Beclin-1 and Bcl-2, release of Beclin-1, and autophagy stimulation [36, 37]. Some data revealed the inhibitory effect of TQ on autophagy on specific tumor cells such as glioblastoma cells. The inhibition of autophagic flux can blunt the formation of autophagolysosomes and the activation of Cathepsin, resulting in the activation of Caspase-independent apoptotic changes [38]. In case the overstimulation of autophagy occurs, other death mechanisms such as apoptosis can be activated. For instance, TQ increases the apoptotic changes in oral cancer cells via activation of Caspase 9 in an LC3-II-dependent manner [39]. Apoptotic death and inhibition of pancreatic cancer stem cells were unveiled by TQ via Notch1 and PI3K/Akt/mTOR-regulated autophagy signaling pathways [40]. These features indicate that overstimulation and inhibition of autophagy can alter the dynamic growth of cancer cells via apoptosis-related mechanisms. It was suggested that TQ can promote autophagic cell death in CPT-11-R LoVo colon cancer cells by mitochondrial membrane injury, and activation of stress-related kinases such as JNK and p38 [41]. The Wnt/β-catenin pathway signaling pathway is a critical molecular axis in the progression of tumor mass, and metastasis to remote sites [42]. Here, we found that TQ effects were similar to the groups that received the Wnt3a blocker. TQ alone or in combination with LGK974 can inhibit the tumorigenic properties of human adenocarcinoma cells. The present work faces some limitations that need further investigation. The inhibitory effects of TQ were assessed on colorectal adenocarcinoma HT-29 cells. The detrimental/beneficial effects of TQ on healthy intestinal epithelial cells should be studied using similar concentrations. Despite the tumoricidal properties of TQ on different cancer cell types, TQ lacks cytotoxicity in normal cells [43]. One reason would be that TQ can reach to subcellular compartment and regulate the activity of various kinases and transcription factors associated with tumorigenesis [43].

## Conclusion

According to data from the current study, it is suggested that TQ can promote the tumoricidal properties of human adenocarcinoma HT-29 cells by the modulation of mitophagy and autophagy mechanisms. These features coincide with the accumulation of intracellular activity of fluorochromes and the reduction of migration rate. Future studies should focus on the detection of several underlying mechanisms with tumoricidal properties after being treated with TQ.

### Electronic supplementary material

Below is the link to the electronic supplementary material.

Supplementary Material 1

Supplementary Material 2

Supplementary Material 3

Supplementary Material 4

## Data Availability

Data will be available from corresponding authors upon reasonable request.

## References

[1] Saraiva MR, Rosa I, Claro I (2023). Early-onset colorectal cancer: a review of current knowledge. World J Gastroenterol.

[2] Xi Y, Xu P (2021). Global colorectal cancer burden in 2020 and projections to 2040. Translational Oncol.

[3] Burada F (2015). Autophagy in colorectal cancer: an important switch from physiology to pathology. World J Gastrointest Oncol.

[4] Bagi HM et al. Interplay between exosomes and autophagy machinery in pain management: state of the art. Neurobiol Pain, 2022: p. 100095. 10.1016/j.ynpai.2022.100095 PMC9198378 35720640

[5] Xia H, Green DR, Zou W (2021). Autophagy in tumour immunity and therapy. Nat Rev Cancer.

[6] Qiang L et al. Deconvoluting the complexity of autophagy in colorectal cancer: from crucial pathways to targeted therapies. Front Oncol, 2022. 12. 10.3389/fonc.2022.1007509 PMC9510924 36172152

[7] Wang S (2022). piR-823 inhibits cell apoptosis via modulating mitophagy by binding to PINK1 in colorectal cancer. Cell Death Dis.

[8] Zhang Y, Wang X (2020). Targeting the Wnt/β-catenin signaling pathway in cancer. J Hematol Oncol.

[9] Turcios L (2019). Autophagic flux modulation by Wnt/β-catenin pathway inhibition in hepatocellular carcinoma. PLoS ONE.

[10] Pérez-Plasencia C (2020). Interplay between autophagy and Wnt/β-catenin signaling in cancer: therapeutic potential through drug repositioning. Front Oncol.

[11] Zhang L, Shay JW. Multiple roles of APC and its therapeutic implications in colorectal cancer. JNCI: J Natl Cancer Inst, 2017. 109(8). 10.1093/jnci/djw332 PMC5963831 28423402

[12] Haque A, Brazeau D, Amin AR (2021). Perspectives on natural compounds in chemoprevention and treatment of cancer: an update with new promising compounds. Eur J Cancer.

[13] Du Y-X (2023). Natural compounds targeting the Autophagy Pathway in the treatment of Colorectal Cancer. Int J Mol Sci.

[14] Ali SR et al. Black seeds (Nigella Satival.)-kalonji: a brief review of its anti-cancer and anti-tumour qualities. Hamdard Medicus, 2022. 65(1).

[15] Gali-Muhtasib H (2008). Thymoquinone reduces mouse colon tumor cell invasion and inhibits tumor growth in murine colon cancer models. J Cell Mol Med.

[16] Zhang L, Bai Y, Yang Y (2016). Thymoquinone chemosensitizes colon cancer cells through inhibition of NF–κB. Oncol Lett.

[17] Haiaty S (2021). Thymoquinone inhibited vasculogenic capacity and promoted mesenchymal-epithelial transition of human breast cancer stem cells. BMC Complement Med Ther.

[18] Farsiabi R (2023). Evaluation of the effects of Thymoquinone on oxidative stress in A549 Lung Cancer Cell line. Middle East J Cancer.

[19] Anand U, et al. Cancer chemotherapy and beyond: current status, drug candidates, associated risks and progress in targeted therapeutics. Genes & Diseases; 2022. 10.1016/j.gendis.2022.02.007 PMC10310991 37397557

[20] van den Boogaard WM, Komninos DS, Vermeij WP (2022). Chemotherapy side-effects: not all DNA damage is equal. Cancers.

[21] Bukowski K, Kciuk M, Kontek R (2020). Mechanisms of multidrug resistance in cancer chemotherapy. Int J Mol Sci.

[22] Pan MH (2011). Molecular mechanisms for chemoprevention of colorectal cancer by natural dietary compounds. Mol Nutr Food Res.

[23] Amin AR (2009). Perspectives for cancer prevention with natural compounds. J Clin Oncol.

[24] Hsu S-C, Chung J-G (2012). Anticancer potential of emodin. BioMedicine.

[25] AbuKhader MM (2013). Thymoquinone in the clinical treatment of cancer: fact or fiction?. Pharmacogn Rev.

[26] Almajali B (2021). Thymoquinone, as a novel therapeutic candidate of cancers. Pharmaceuticals.

[27] Goyal SN (2017). Therapeutic potential and pharmaceutical development of thymoquinone: a multitargeted molecule of natural origin. Front Pharmacol.

[28] Koka PS (2010). Studies on molecular mechanisms of growth inhibitory effects of thymoquinone against prostate cancer cells: role of reactive oxygen species. Experimental Biology Med.

[29] Zhao Z (2023). Advances in research on the relationship between thymoquinone and pancreatic cancer. Front Oncol.

[30] Dirican A (2015). Novel combination of docetaxel and thymoquinone induces synergistic cytotoxicity and apoptosis in DU-145 human prostate cancer cells by modulating PI3K–AKT pathway. Clin Transl Oncol.

[31] López-Méndez TB (2023). Nanomedicine for autophagy modulation in cancer therapy: a clinical perspective. Cell Bioscience.

[32] Li J (2009). Inhibition of autophagy by 3-MA enhances the effect of 5-FU-induced apoptosis in colon cancer cells. Ann Surg Oncol.

[33] Carew JS (2010). Autophagy inhibition enhances vorinostat-induced apoptosis via ubiquitinated protein accumulation. J Cell Mol Med.

[34] Li J-L, Han S-L, Fan X (2011). Modulating autophagy: a strategy for cancer therapy. Chin J Cancer.

[35] İLHAN S, Ferdi O (2021). Induction of autophagic cell death by thymoquinone in docetaxel resistant prostate cancer cells. Duzce Med J.

[36] Wei Y (2008). JNK1-mediated phosphorylation of Bcl-2 regulates starvation-induced autophagy. Mol Cell.

[37] Zalckvar E (2009). DAP-kinase‐mediated phosphorylation on the BH3 domain of beclin 1 promotes dissociation of beclin 1 from Bcl‐XL and induction of autophagy. EMBO Rep.

[38] Racoma IO (2013). Thymoquinone inhibits autophagy and induces cathepsin-mediated, caspase-independent cell death in glioblastoma cells. PLoS ONE.

[39] Chu S-C (2014). Thymoquinone induces cell death in human squamous carcinoma cells via caspase activation-dependent apoptosis and LC3-II activation-dependent autophagy. PLoS ONE.

[40] Mu G-g (2015). Thymoquinone pretreatment overcomes the insensitivity and potentiates the antitumor effect of gemcitabine through abrogation of Notch1, PI3K/Akt/mTOR regulated signaling pathways in pancreatic cancer. Dig Dis Sci.

[41] Chen M-C (2015). Thymoquinone induces caspase-independent, autophagic cell death in CPT-11-resistant lovo colon cancer via mitochondrial dysfunction and activation of JNK and p38. J Agric Food Chem.

[42] Zhang Y, Wang X (2020). Targeting the Wnt/β-catenin signaling pathway in cancer. J Hematol Oncol.

[43] Homayoonfal M, Asemi Z, Yousefi B (2022). Potential anticancer properties and mechanisms of thymoquinone in osteosarcoma and bone metastasis. Cell Mol Biol Lett.

