# Supplementary figures and images for: Tumoricidal properties of thymoquinone on human colorectal adenocarcinoma cells via the modulation of autophagy
Source: BMC Complement Med Ther. 2024 Mar 26;24:132. doi: 10.1186/s12906-024-04432-2 (PMC10964622; doi:10.1186/s12906-024-04432-2)

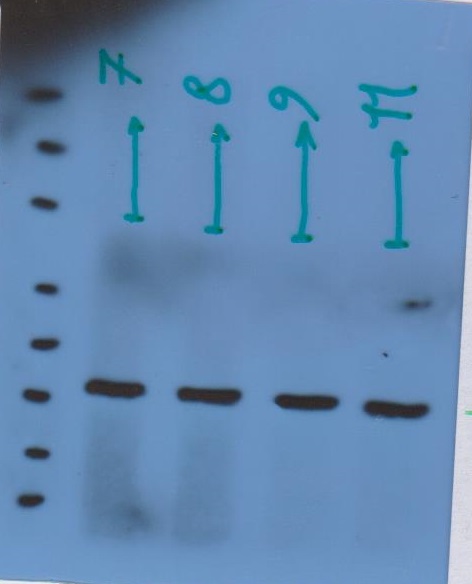

Supplement: Supplementary file 1 — Supplementary Material 1 [file 12906_2024_4432_MOESM1_ESM.jpg]

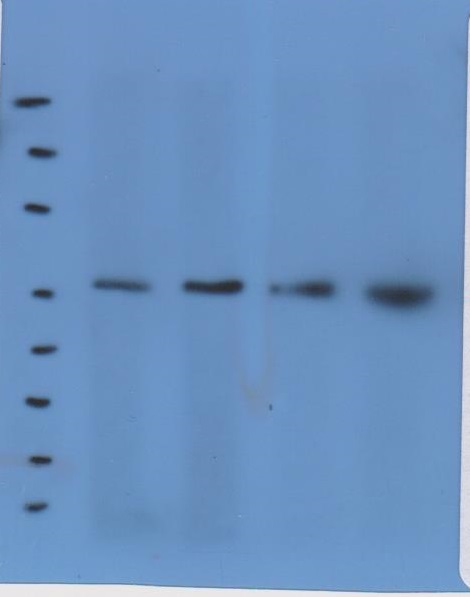

Supplement: Supplementary file 2 — Supplementary Material 2 [file 12906_2024_4432_MOESM2_ESM.jpg]

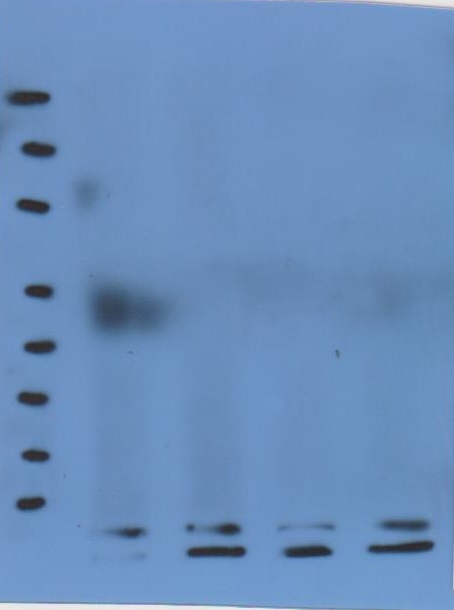

Supplement: Supplementary file 3 — Supplementary Material 3 [file 12906_2024_4432_MOESM3_ESM.jpg]

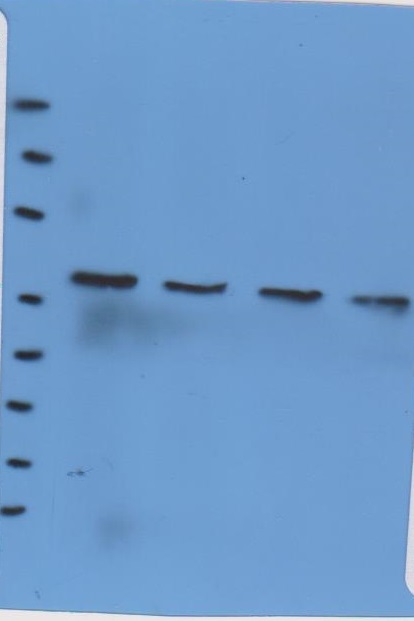

Supplement: Supplementary file 4 — Supplementary Material 4 [file 12906_2024_4432_MOESM4_ESM.jpg]
